# UPLC–ESI-PDA–Ms^n^ Profiling Of Phenolics Involved In Biological Activities Of The Medicinal Plant *Halocnemum Strobilaceum *(Pall*.*)

**Published:** 2019

**Authors:** Heba Handoussa, Walid AbdAllah, Mona AbdelMohsen

**Affiliations:** a *Department of Pharmaceutical Biology, Faculty of Pharmacy and Biotechnology, German University in Cairo GUC, Egypt.*; b *Department of Phytochemistry, National Research Centre, 33 El Buhouth St. (Former El Tahrir St.), 12622-Dokki, Giza, Egypt.*

**Keywords:** Halocnemum strobilaceum, Phenolics, UPLC/PDA/ESI/MSn, Anticancer, Antioxidant

## Abstract

*Halocnemum strobilaceum* is a halophyte present in the humid and arid bioclimatic regions of Egypt. The current study aimed at UPLC/PDA/ESI-MS^n ^qualitative chemical profiling of the phyto-constituents underlining both antioxidant and cytotoxic activities of the bio-active fraction in comparison with the other fractions. It resulted in detection of several related compounds to prenylated flavonol icariin as a first report in this species*.* Results showed that ethyl acetate exhibits an appreciable antioxidant activity using *in-vitro* DPPH assay with percentage of 82.35% and remarkable anticancer capacity against most common types of cancer in Egypt; breast (MCF-7), human prostate (PC-3) cancer cell lines, and human lung carcinoma (A-549) with IC_50_ 43.1± 2 µg/mL, 115±5 µg/ml, and 53.3±3 µg/mL respectively. These findings point out the appropriate solvent for extraction of the bio-phenolics with this halophyte which is a considerable source of remarkable potential secondary metabolites that exhibit original and interesting anticancer capacity.

## Introduction

Natural antioxidants are mainly secondary metabolites synthesized by plants such as phenolic acids, flavonoids, and terpenoids, to be involved in sustaining growth or protecting the more vulnerable cell constituents under adverse environment, exemplified as herbaceous halophytic plants which live in harsh environments; high temperature and wide variety in salinity levels. Thus, grow in coastal regions, salt marshes and mudflats to dry deserts. These plants may have unique variety of compounds not found in other species, or in higher concentrations according to stress response mechanisms (1). They develop adaptive responses through a powerful antioxidant system, including synthesis of several bioactive compounds as secondary metabolites which display potent biological activities (2).

In Egypt, a considerable diversity of plant species with multiple interests including therapeutic practices has not been subjected to chemical investigations. Among them, is *Halocnemum strobilaceum *which is a succulent halophyte, distributed along the Mediterranean coast. It usually grows in pure sands, either in circular or as irregular patches on flat tidal mud, or shoreline sand bars, forming high hillocks, with a relatively shallow root system (3).

The current study aimed at estimating the phenolic content, evaluating the potencyof different fractions (hexane, ethyl acetate, butanol and water) of Halocnemum strobilaceum to act as antioxidant and cytotoxic halophyte.Furthemore, a rapid comprehensive evaluation using UPLC/PDA/ESI-MSn qualitative chemical profiling of the most bioactive fraction was applied to characterize the phyto-constituents underlining these activities. Human breast (MCF-7), prostate (PC-3) cancer cell lines and human lung carcinoma (A-549) were selectively studied to represent the most common types of cancer especially in Egypt.

## Experimental

*Plant material, extraction and fractionation*

*Halocnemum strobiolaceum *(Pall.), Family Chenopodiaceae was collected from natural populations site in the Mediterranean coastal zone away from human activities, Egypt, during Spring season. It was authenticated by Assistant Prof. Dr. M. El-Gebaly, taxonomist, National Research Centre (NRC), Giza, Egypt, where a voucher specimen was deposited. 

The aerial parts were air dried at ambient temperature; 500 g of the dried sample was extracted twice with 90% methanol at room temperature for 48 h. After filtration, the combined extracts were concentrated using a rotary evaporator under reduced pressure at 50 ºC. The residue obtained, was suspended in water and fractionated with several solvents with increasing polarity. Each of the fractions; yielded as follows *n*-hexane (5gms), ethyl acetate (12gms) and *n*-butanol (6gms) that were dried using rotary evaporator and were freeze-dried, then the residues were resuspended in dimethyl sulphoxide (DMSO) before testing.

*Chemical reagents*

Folin-Ciocalteu reagent was obtained from Loba-Chemie (Mumbai, India), sodium carbonate anhydrous (Na_2_CO_3_), gallic acid, 2, 2- diphenyl-1-picrylhydrazyl (DPPH), and Trolox (6-hydroxy-2,5,7,8-tetramethyl chroman-2-carboxylic acid) were purchased from Aldrich Chem. Co. All the reagents used were of HPLC grade. 

*Total phenolic content*

Total phenolic content was determined as described by (4). An aliquot of the diluted sample extract was mixed with 0.5 mL distilled water and 0.125 mL of Folin-Ciocalteu reagent. After 6 min, 1.25 mL of 7% Na_2_CO_3 _was added and the solution was adjusted to a final volume of 3 mL with distilled water. The absorbance of the resulting solution was recorded at 760 nm, after incubation for 90 min. Total phenolic content was expressed as milligram gallic acid equivalent per gram of dry weight (mg GAE/g DW) through a calibration curve with gallic acid. All samples were analyzed in 

triplicates.

*DPPH radical scavenging activity*

The free radical scavenging activity (RSA) of all fractions against DPPH was determined as described by (5). 1 mL of different concentrations of the samples were added to 0.25 mL of methanolic solution of DPPH (0.2 mmol/L) and allowed to react in darkness for 30 min. The absorbance was measured against a blank at 517 nm. Trolox was used as a reference. The assay was carried in triplicate and the percentage of inhibition was calculated using the following formula: 

*Cell culture*

All materials and reagents for the cell cultures were purchased from Lonza (Verviers, Belgium). Breast carcinoma (MCF-7) and prostate carcinoma (PC-3) were obtained from VACSERA Tissue Culture Unit, human cancer cell lines of lung carcinoma (A-549) (ATCC, Rockville, MD). The cell lines were maintained as monolayer culture in Dulbecco’s modified Eagle’s medium (DMEM) supplemented with 10% FBS, 4 mM L-glutamine, 100 U/mL penicillin, and 100 µg/mL streptomycin sulfate. The monolayers were passaged at 70–90% confluence using a trypsin-EDTA solution. All the cells were incubated in a humidified atmosphere at 37 ºC with 5% CO_2_.

*Cytotoxicity assay*

Cytotoxicity studies were performed using a modified MTT (3-[4, 5]-2, 5-diphenyltetrazolium bromide) assay (6). Exponentially growing tumor cells were seeded at a density of 5x10^4 ^cell/well in Corning® 96-well tissue culture plates, incubated for 24 h. 100 µL of increasing concentrations of the fractions were added in culture medium (final DMSO concentration in medium = 0.5 % v/v). About six vehicle controls with media or 0.5 % DMSO were run for each 96 well plate as a control. After 48 h of incubation, MTT solution in PBS (5 mg/mL) was added to each well, including the untreated control, after which the incubation was resumed for further 4 h. The formation of intracellular formazan crystals (mitochondrial reduction product of MTT) was confirmed by a phase contrast microscopic examination. Then the medium was removed, and 50 µL of DMSO was added to each well to dissolve formed formazan crystals with shacking for 10 min (200 rpm). Dissolved crystals were quantified by reading the absorbance at 590 nm (OD) on a microplate reader (Sunrise™ microplate reader, Tecan Austria Gmbh, Grödig, Austria). The cell viability was determined by comparing the average OD values of the control wells with those of the samples (quadrate to octuplet treatments), both represented as % viability [control (0.1% DMSO only) = 100%]. 

$\mathrm{formula}:\mathrm{RSA\%}=\frac{{\mathrm{Blank}}_{\left(\mathrm{abs}\right)}-{\mathrm{Sample}}_{\left(\mathrm{abs}\right)}}{{\mathrm{Blank}}_{\left(\mathrm{abs}\right)}}\times 100$

Equ. 1

*UPLC–ESI-PDA–MS* ^n^ *profiling*

The chromatographic analysis was performed on UPLC Agilent 1200 series instrument using column; Gemini 3 mm C18 110A° from Phenomenex with dimensions 100 X 1 mm i.d., protected with RP C18 100 A° guard column with dimensions (5 mm X 300 mm i.d., 5 mm). The mobile phase consisted of two solvents; 2% acetic acid (A) and 90% MeOH (B) at a flow rate of 0.5 mL/min. The sample was dissolved in 5% MeOH and 2% acetic acid while the sample injection volume was 10 µL. A Fourier transform ion cyclotron resonance mass analyzer was used equipped with an electrospray ionization (ESI) system. X-calibur® software was used to control the system. Detection was performed in the negative ion mode applying a capillary voltage of 36 V and a temperature of 275 °C. The API source voltage was adjusted to 5 kV, and the desolvation temperature to 275 °C. Nitrogen was used as a nebulizing gas with a flow adjusted to 15 L/min. The full mass scan covered the mass range from 150 to 2,000 *m/z* with resolution up to 100,000 (7).

*Statistical analysis*

The IC_50_ values (concentration of sample causing 50% loss of intact cells of the vehicle control) were presented as mean values ± (SD) and were calculated using the concentration-response curve fit to the non-linear regression model using GraphPad Prism® v6.0 software (GraphPad Software Inc., San Diego, CA, USA). Values of *p *< 0.05 were assumed as statistically significant.

## Results

Several studies showed that phenolics constitute the main powerful antioxidant compounds (8, 9). As a result, total phenolics, and antioxidant activity of *Halocnemum strobiolaceum *extracts were assessed. Results showed that among the three solvent fractions (hexane, ethyl acetate, butanol), the ethyl acetate fraction exhibited an apperciable amount of phenolic content (29.42 mg GAE/g DW), followed by butanol of 18.02 mg GAE/g DW. Moreover, the antioxidant activity of these fractions was assessed *in-vitro* using radical scavenging capacity (DPPH) assay. The results indicated that ethyl acetate fraction showed the highest antioxidant activity with a percentage of (82.35%) followed by butanol extract (65.43%), while in lower solvent polarity; water and hexane extracts, this activity was less significant, the assay was done in comparison to standard antioxidant, Trolox (95.73%). These results manifest clearly the influence of the solvent on the extractability of strong antioxidant compounds, including phenolics, which has been previously reported (10). 

*Evaluation of cytotoxicity against tumor cell lines*

Oxidative stress is known among the main causes of cancer-related death. In recent years, natural derived-bioactive substances that are capable to prevent or suppress cancer progress have received great attention in cancer chemo preventive approaches (11). Phenolic compounds which are powerful antioxidants are known to have antiproliferative activities against most cancer cell lines (12). Thus, the cytotoxic activity of *Hs *phenolic fractions (ethyl acetate, butanol) was evaluated against three human tumor cells originating from breast carcinoma (MCF-7), prostate carcinoma (PC-3), and lung carcinoma (A-549), based on MTT reduction assay. The IC_50_ values obtained are shown in (Table 1).

**Table 1:** Cytotoxic activity of *Halocnemum strobiolaceum *extracts against three tumor cell lines

**Extracts**	**IC** **50 (µg/mL)**		
	**A-549**	**MCF-7**	**PC-3**
Ethyl acetate	53.3 ± 3	43.1 ± 2	115 ± 5
Butanol	›200	›200	›200
Doxorubicin HCL	0.95	0.35	3.68

**Table 2:** Peak assignments using UPLC/PDA/ESI-MS-MS of metabolites detected in ethyl acetate (EA) fraction of *Halocnemum strobilaceum *L. from (negative mode)

**Peak # (Compound)**	**Identified Compounds**	**Retention time (min)**	**UV-Vis (λmax)**	**[M-H]** **- ** **(** ***m/z*** **)**	**Fragment ions (** ***m/z*** **)**	**Reference**
Hs1	Sinapic acid hexoside	1.09	325	385	223,179	(15)
Hs2	Quercetin-*O*-gluc-*O*-rhamnose	1.16	256, 352	593	463,301,179	(13)
Hs3	Quercetin-O-glucoside	1.13	255, 354	463	301,179	(13)
Hs4	(Iso)rhemetin-*O*- hexoside	1.46	268, 342	477	315,300,283,271,151	(13)
Hs5	(Iso)rhamnetin-3-O-rutinoside	1.5	268, 342	623	314,315,300	(18)
Hs6	Quercetin rhamnoside	5.9	254, 353	447	315, 301,179	(15)
Hs7	Phloretin-2-O-glucosyl hexoside	7.5	226,286	597	298	(18)
Hs8	Hydroxyterpenylic acid	7.8	237	187	181,145	(15)
Hs9	Quercetin pentosyl-hexoside isomer	8.2	253, 356	595	301,179	(17)
Hs10	Quercetin pentosyl-hexoside	8.76	254, 356	595	301,179	(17)
Hs11	Rutin	9.06	256, 352	609	301,179	(7)
Hs12	Methyl gallate hexoside	10.6	278	345	331	(15)
Hs13	Galloyl-hexoside	10.8	278	331	179	(21)
Hs14	Rhamnazin	11.2	256, 352	329	314,299,271	(19)
Hs15	Demethylanhydroicaritin-3-Orha-(1–2)-glu	13.33	270,360	661	499,352,325,311	(30)
Hs16	Demethylanhydroicaritin-3-O-rha-(1–2)-rha	14.36	269,364	645	499,352,325,311	(30)
Hs17	Demethylanhydroicaritin-3-O rha-(1–2)-OAc	14.7	270,360	763	631,325,311	(30)
Hs18	Demethylanhydroicaritin-3-O-rha-(1–2)-xyl	15.4	271,360	631	499, 352,325,311	(30)
Hs19	Anhydroicaritin derivative	22.69	270,362	453	352,325,311	(30)
Hs20	Hydroxyicartine glycosides derivative	23.32	268, 360	637	352,325,311	(30)

*Metabolites’ Profiling of active fraction*

This study aimed at fingerprinting of the phyto-constituents (Table 2) within the bioactive ethyl acetate fraction, which showed cytotoxic activity using ESI Accurate-Mass -PDA-MS^n^ approach. The characterization revealed the presence of phenolic acids, flavonoids, and several glycosides. Several quercetin derivatives were detected; as in (**Hs3**) having its deprotonated molecule of [M−H]^− ^at *m/z* 463 with fragments at *m/z* 301 [M−162−H]^ − ^ as well as the diagnostic product ion peak [M−H−CO]^− ^appeared at *m/z* 271 typical to flavon-3-*O*-monoglycoside and at *m/z* 179 from the RDA of ring A. Such fragments confirmed the presence of quercetin hexoside (13) that was previously isolated from the same species (14).

(**Hs2**) and (**Hs6**) represent flavonoid quercetin with rhmonosyl residue which was in agreement with the reported data of quercetin-*O*-gluc-*O*-rhamnose (13) and quercetin rhamnoside (15), which was isolated from this plant as reported in (16). Furthermore, two Peaks (**Hs9**&**Hs10**) appeared on different retention times and were characterized as quercetin-pentoside-hexoside and its isomer; both having parent ion of [M−H]^−^ at *m*/*z *595 and fragment ions at *m*/*z *433 and 301 that were obtained due to a sequential loss of hexose [M − H − hexose] and pentose [M − H − hexose − pentose] moieties respectively (17), similarly, quercetin-rutinoside (rutin) was recognized as peak (**Hs11**) by molecular ion [M–H]^−^ of *m/z* 609 with characteristic MS/MS fragment ions pattern which is consistent with Handoussa *et al*., 2013 (7).

Methylated flavonol derivatives have been shown within the ethyl acetate fraction as in peaks; (**Hs4**) identified as (iso) rhamnetin-3-*O*-glucoside which was previously isolated from the same halophyte (13), its identity was confirmed by the fragments; *m/z* 315 and *m/z* 301 as isorhamnetin, and demethylated aglycone, respectively. Besides, peak (**Hs5**) recognized as (iso) rhamnetin-3-*O*-rutinoside that produced a [M–H]^–^ at *m/z* 623, in which MS–MS spectrum showed two predominant fragments at *m/z* 315 [M–H–308]^−^ and 314 [M–H–308]^−^ were observed. The loss of 308 Da (162 + 146) indicated that a hexose and a deoxyhexose linked at the same position of the aglycone. The substituent position of 3-OH was suggested by the intensity of [M–H–308]^−^• which is higher than that of [M–H–308]^– ^(18). Isorhamnetin was confirmed as aglycone by the characteristic fragments; *m/z* 300, *m/z* 271, *m/z* 255 and *m/z* 227) (18). Furthermore, Peak (**Hs14**) which was characterized as rhamnazin; owing to its [M–H] ^–^ at *m/z *329, and the fragment ions *m/z* 314 [M−H−CH_3_]^ −^, *m/z* 299 [M−H−2CH_3_]^−^, and *m/z* 271 [M−H−CO−2CH_3_]^ – ^(19).

Additionally, Six prenylated flavonol glycoside (**Hs15 - Hs20**) were detected as a first report for this species related to icariin moiety. Peaks (**Hs15 - Hs20**) are having deprotonated molecule [M−H]^−^ of ions at *m/z* 661, *m/z* 645, *m/z* 763, *m/z* 631, *m/z* 453 and *m/z* 637 respectively, and all of these deprotonated ions produced the ions at *m/z* 499, *m/z* 481,* m/z *352, and *m/z* 323 in their MS^n^ spectra. The [Y_0_−HCO−H] ^•− ^ion at *m/z* 323 indicated the linkage of diglycosides at the C-3 position of aglycone. The other characteristic ion at *m/z* 481 revealed the outer sugar of 3-*O*-diglycosides attaching to the C-2 or C-4 position of rhamnose. According to their relative contents and the reported literature, these peaks were tentatively characterized as demethylanhydroicaritin-3-*O*-rha-(1–2)-glu, demethylanhydroicaritin-3-*O*-rha-(1–2)-rha, demethylanhydroicaritin-3-*O*-rha-(1–2)-OAc, demethylanhydroicaritin-3-*O*-rha-(1–2)-xyl, anhydroicaritin derivative and hydroxyicartine glycosides derivative respectively.

## Discussion

Epidemiological studies have demonstrated that populations consuming high levels of plant derived foods have low incidence rates of various cancers. Recent findings implicate the protective role of plant phenolics, in increasing the average life span and lowering the incidence of a wide variety of human cancer. Both monophenolic and polyphenolic compounds from different plants have been shown to inhibit or attenuate the initiation, progression and spread of cancers in cells *in-vitro* and *in-vivo* studies, in a process called radical scavenging to carry them away, so they offer opportunities for innovation in drug discovery (20).

The enduring relationship between antioxidant capacity and carcinopreventive agents has been proven within the current study as the ethyl acetate fraction with its enriched content of different polyphenolic moieties showing a considerable antioxidant capacity with a percentage of 82.35% and thus preventing oxidative stress which leads to damage of DNA, induction of apoptosis, and inhibition of growth and proliferation of cancer cells (21).

In this study, phytochemical investigation of the bioactive fraction derived from *Hs *was performed by UPLC/PDA/ESI-MS^n ^revealinng the presence of several phenolic classes. Interestingly, icaritin derived compounds were found to possess the major peaks’ area (25%) of the whole bioactive ethyl-acetate fraction. It was reported that icaritin which is a structural analogue to genistein and daidzein, leads to increment of the estrogen receptor-regulated progesterone receptor and PS2 mRNA levels in MCF-7 cells (22). Icaritin induced cell growth inhibition, G1 arrest, and mitochondrial transmembrane potential drop in human prostate carcinoma PC-3 cells, besides, it can induce cell apoptosis accompanied by activation of caspases as evidenced by the cleavage of endogenous substrate Poly (ADP-ribose) polymerase (PARP) (23). This activity could be further correlated to the capability of this prenylated flavonol glycoside to induce cell cycle arrest at the G2/M phase accompanied by a down-regulation of the expression levels of the G2/M regulatory proteins such as cyclin B, cdc2, and cdc25C (24). In addition, inhibit transcription of key androgen receptor regulated genes, such as KLK3 (PSA) and TMPRSS2 and induce apoptosis in both androgen-sensitive and castration-resistant prostate cancer cells. The potent effect of ethyl acetate on A-549 is consistent with (25) which proved that icaritin is an effective inhibitor by inducing S phase arrest and apoptosis in human lung carcinoma A549 cells.

The metabolites’ profiling within the fraction highlighted the presence of quercetin with all its isoforms as free aglycone or in glycosidic form. Several publications investigated the mechanism underlying the antiproliferative effect of quercetin as it plays a crucial role in limiting the cancerous cell proliferation as was reported to induce cell cycle arrest during either G1 or G2/M and decreasing expression of Bax gene, reduced apoptotic index, increased cell viability and antiproliferative effect on MCF-7 cells (26), besides the apoptotic activity in different breast cancer cells through suppression of Twist via p38MAPK pathway (27). Similar studies were performed on PC-3 resulted in reduction of pSTAT3, pERK1/2, pAKT protein levels, induction of the oxidative stress, and generation of reactive oxygen species (28), and also, lowering vascular endothelial growth factor protein and mRNA expression, microvascular density and proliferation on PC-3 (28). Moreover, it stimulated cell proliferation and increased total antioxidant capacity of A549 cells and triggered BCL2/BAX*-*mediated apoptosis, as well as necrosis and mitotic catastrophe, and inhibited the migratory potential of A-549 lung cancer cells (29). 

The current study sheds light on the promising effect of *Halocnemum strobilaceum* and strengthens the potential of its usage as a potent antioxidant, anticancer and chemopreventive agent with a synergistic activity between all its phytochemicals to give utmost protection level against generation of reactive oxygen species and further against cancer.

## Conclusion

An UPLC/PDA/ESI-MS^n^ based metabolomics analysis of the bioactive ethyl acetate fraction against cancer cell lines; PC3, MCF-7, and A549 revealed that it is rich in flavonoid glycosides with quercetin, isorhamnetin, and icaritin moieties. These results suggest that ethyl acetate fraction derived from *Halocnemum strobilaceum* might act as a rich source of natural antioxidants with potent anticancer activity.
